# Two successful pregnancies and first use of empagliflozin during pregnancy in glycogen storage disease type Ib

**DOI:** 10.1002/jmd2.12295

**Published:** 2022-05-18

**Authors:** Sarah Catharina Grünert, Stefanie Rosenbaum‐Fabian, Anke Schumann, Anne‐Christine Selbitz, Waltraut Merz, Andrea Gieselmann, Ute Spiekerkoetter

**Affiliations:** ^1^ Department of General Paediatrics, Adolescent Medicine and Neonatology Medical Centre‐University of Freiburg, Faculty of Medicine Freiburg Germany; ^2^ Institute of Experimental Hematology and Transfusion Medicine University Hospital Bonn Bonn Germany; ^3^ Department of Obstetrics and Prenatal Medicine University Bonn Medical School Bonn Germany; ^4^ Department of Pediatric Cardiology University Hospital Bonn Bonn Germany

**Keywords:** empagliflozin, G‐CSF, glucose‐6‐phosphate transporter, glycogen storage disease type Ib, GSD Ib, pregnancy, SLC37A4

## Abstract

Glycogen storage disease type Ib (GSD Ib) is caused by biallelic variants in *SLC37A4*. GSD Ib is characterized by hepatomegaly, recurrent hypoglycemia, neutropenia, and neutrophil dysfunction. Only seven pregnancies in four women with GSD Ib have been reported so far. We report on two further successful pregnancies in two patients with GSD Ib. One of these pregnancies was managed with empagliflozin, an SGLT2 inhibitor, repurposed for the treatment of neutropenia in GSD Ib. Both pregnancies were unremarkable and resulted in healthy offspring. Gestational care and pre‐ and perinatal management in GSD Ib are challenging and require close interdisciplinary metabolic and obstetric monitoring. In our patient, the use of empagliflozin during pregnancy was successful in the prevention of neutropenic symptoms and infections and enabled good wound healing after Cesarean section, while no adverse effects were observed.

**Synopsis**

The use of empagliflozin in GSD Ib during pregnancy was safe and resulted in a healthy child.

## 1 INTRODUCTION

Glycogen storage disease type Ib (GSD Ib, OMIM # 232220) is an ultrarare disorder of glycogen metabolism caused by biallelic mutations in the *SLC37A4* gene.^1^ These result in deficiency of the endoplasmatic glucose‐6‐phosphate transporter (G6PT) that transfers glucose‐6‐phosphate into the endoplasmic reticulum for hydrolysis by glucose‐6‐phosphatase. The latter enzyme catalyzes the final reaction of both glycogenolysis and gluconeogenesis. Therefore, GSD Ib results in impaired endogenous glucose production. Accumulating glucose‐6‐phosphate is consequently shunted into various pathways leading to hyperuricemia, hypertriglyceridemia and hyperlactatemia.^2^

The incidence of GSD Ib is approximately 1:500000–1:1.000000^3^. The clinical phenotype is characterized by severe fasting hypoglycemia, hepatomegaly, and neutropenia with neutrophil dysfunction, associated with Crohn‐like inflammatory bowel disease, mucosal ulcerations and recurrent infections.^1^, ^4^ Before the introduction of granulocyte colony‐stimulating factor (G‐CSF) for the treatment of neutropenia in the 1980s, only few individuals with GSD Ib survived into adulthood, while nowadays, with improved treatment strategies, most patients reach adulthood and the child‐bearing age. The recent elucidation of the mechanisms of neutropenia in GSD Ib and the new treatment option with SGLT2 inhibitors^5^ are likely to further ameliorate the long‐term prognosis of patients with GSD Ib. To our knowledge, only seven successful pregnancies in four women with GSD Ib have been reported so far.^6^, ^7^ We herein describe the course and management of two successful pregnancies in two further women with GSD type Ib. We also report the first use of empaglifozin in a GSD Ib patient during pregnancy with favorable outcome.

## 2 CASE PRESENTATIONS

### 2.1 Patient 1

The patient, a 34‐year‐old woman, was diagnosed with GSD Ib at the age of 7 months. Records of her clinical course throughout childhood were not available. Due to neutropenia with recurrent infections, she has been on G‐CSF therapy since the age of 12 years. She had never developed inflammatory bowel disease. Prior to pregnancy, she was in good metabolic control (normal transaminase activities, triglycerides <200 mg/dl, uric acid within normal range) and mild hepatosplenomegaly. Euglycemia was maintained by regular cornstarch administration, and the patient used continuous glucose monitoring.

Pregnancy became known in the 6th week of gestation. Details on pregnancy and birth are shown in Table 1; information on dietary management during pregnancy is shown in Table 2. Apart from G‐CSF, vitamins and L‐thyroxine, she was not on any medication. Early pregnancy was unremarkable with no nausea or vomiting reported. However, blood glucose concentrations were fluctuating with recurrent mild hypoglycemia requiring oral glucose administration. At the beginning of the second trimester, she suffered from abdominal pain and heartburn. From the 20th week of gestation, the metabolic stability improved. In the 22nd week of gestation, dental surgery had to be performed and the patient received antibiotic treatment for 1 week. The G‐CSF dose was transiently increased. At 30 weeks of gestation, abdominal ultrasound was performed confirming the previously known hepatosplenomegaly (liver 14.6 cm), but no focal lesions. During the third trimester, the patient reported an increasing energy demand, especially when she was physically active. At the end of pregnancy, she required up to 9 doses of cornstarch per day and regular glucose intake in order to maintain euglycemia during regular physical activity (walking). The total weight gain throughout pregnancy was 17 kg.

**TABLE 1:** Summary of pregnancy outcome and complications in mothers with GSD Ib

Patient	Patient 1	Patient 2
Age at conception	33 years	35 years
G‐CSF (dose)	0.45 μg/kg/day	no
Empagliflozin (dose)	No	20 mg/day
Weight before pregnancy	71.4 kg	50 kg
Weight gain in pregnancy	17 kg	11 kg
Week of delivery	40	37
Mode of delivery	Cesarean section	Cesarean section
Birth weight	3510 g	2940 g
Inflammatory bowel disease	No	diagnosed at the age of 11 years, but no gastrointestinal symptoms
Liver complications/adenomas	None	none
Kidney status	Normal kidney function	Normal kidney function
	No proteinuria	No proteinuria
	No kidney stones	No kidney stones
Inflammatory complications	Dental surgery due to dental infection in week 22 of gestation with antibiotic treatment, impaired wound healing after Cesarean section	none
Laboratory results	Transaminases within normal range throughout pregnancy, triglyceride concentrations max. 2.4 mmol/L neutropenia (neutrophil count <1000/μL)	Transaminases within normal range throughout pregnancy, triglyceride concentrations <2.2 mmol/L normal neutrophil count
Breast feeding	No	Yes

**TABLE 2:** Summary of dietary treatment in 2 women with glycogen storage disease type Ib during pregnancy and breastfeeding

Patient 1	Daytime cornstarch supplements	Nighttime cornstarch	Fasting tolerance/Metabolic stability
First trimester	6–7 doses of about 50 g	2 doses (55 and 35 g)	Abdominal pain and heartburn, increasing carbohydrate demand
Second trimester	6–7 doses of about 50 g	2 doses of up to 55 g	Metabolic instability, abdominal pain and heartburn until 20th week of gestation, almost daily mild hypoglycemias, afterwards significantly stabilized with reduction of cornstarch intake, high tendency to hypoglycemia during dental infection, additional glucose intake necessary
Third trimester	8–9 doses of about 50 g	At the end of pregnancy 3 doses (every 2.5 h) of about 50 g	Increasing carbohydrate demand, especially at the end of pregnancy very short fasting tolerance, additional glucose intake necessary

Patient 2	Daytime cornstarch supplements	Nighttime Glycosade®	Fasting tolerance/Metabolic stability
First trimester	1–2 doses of (30–60 g of uncooked cornstarch per dose)	2–3 doses, within the first weeks average dose of 35–40 g, at the end of the 1st trimester up to 82 g per dose	No nausea or vomiting, rather unstable metabolic situation with short fasting tolerance, increasing carbohydrate demand
Second trimester	1 dose of 40–45 g on most days	2 doses of 82–85 g, 1 dose of 40 g	Metabolic situation stabilized, fasting tolerance about 3 h
Third trimester	1 dose of 45–50 g intermittently	2 doses of 90 g	Short fasting tolerance
Breast feeding	1 dose of 60 g	2 doses of 90 g	Metabolic instability, very short fasting tolerance, meals every 2 h during the day

At 40 + 6 weeks of gestation, Cesarean Section (CS) was performed after unsuccessful induction of labor. The patient received an intravenous glucose‐electrolyte infusion, and the blood glucose level was maintained stable with no documented hypoglycemia. The newborn son had a birth weight of 3510 g with a length of 52 cm. He showed good postnatal adaptation with an Apgar score of 9/10/10. Regular blood glucose measurements within the first 3 days ruled out neonatal hypoglycemia.

Breastfeeding was difficult and discontinued after 4 weeks. Following the delivery, delayed wound healing of the CS scar occurred as well as a panaritium requiring antibiotic treatment. The patient therefore started empagliflozin treatment post delivery, which resulted in normalization of wound healing and normal neutrophil counts. G‐CSF therapy could be stopped 2 weeks after the initiation of empagliflozin.

### 2.2 Patient 2

Patient 2 is a 35‐year‐old woman, who was diagnosed with GSD Ib at the age of 5 months due to hepatomegaly and recurrent hypoglycemia. The diagnosis was made by liver biopsy. She experienced many episodes of gastroenteritis, recurrent skin infections as well as anogenital mucosal lesions during childhood. A Crohn‐like disease was diagnosed at the age of 11 by colonoscopy and elevated calprotectin levels. She never had gastrointestinal symptoms such as diarrhea or abdominal pain. At the age of 13 years, she had rheumatic fever that resulted in severe mitral insufficiency requiring implantation of a mechanical valve at the mitral position. Since then, the patient was on anticoagulant therapy with phenprocoumon. At age 14, she experienced severe respiratory failure. At this time, G‐CSF treatment was started due to neutropenia and recurrent infections. The further course was complicated by a stroke with transient hemiparesis due to air embolism at the age of 27 years. At 29 years, she suffered from kidney stones, while her renal function was always unimpaired. Within the last year before her pregnancy, she had recurrent dental infections, oral mucosal lesions, and a skin abscess. Her liver (20.1 cm) and spleen (12.3 cm) were enlarged, but no adenoma was present. Under continuous glucose monitoring, her metabolic control was good with only rare hypoglycemias. She had normal transaminase activities, triglycerides of 2.1 mmol/L, uric acid of 0.38 mmol/L (normal < 0.36 mmol/L), and no microalbuminuria. She maintained her glucose concentrations by regular meals during the day and continuous maltodextrin infusion via a nasogastric tube during the night. Before pregnancy, she was in an excellent clinical condition and physically very active. Empagliflozin therapy (2 × 10 mg/day) was started at the age of 34 years and led to a normalization of her neutrophil count. G‐CSF therapy could be stopped 4 weeks after the initiation of empagliflozin treatment. Apart from allopurinol, phenprocoumon, and empagliflozin, she had no further medication.

When the patient expressed her wish to become pregnant, she was counseled about the higher risks, particularly due to her cardiac situation with an mechanical mitral valve requiring anticoagulation with phenprocoumon (target INR 2.5–3.5; WHO risk classification III).^8^ The pregnancy became known within the 5th week of gestation. Allopurinol treatment was immediately stopped. Details on pregnancy and birth are shown in Table 1; information on dietary management during pregnancy and breastfeeding is shown in Table 2. Within the first weeks of gestation, the blood glucose concentrations were very unstable, especially in the night. Therefore, the patient administered Glycosade® during the night and additional doses of cornstarch during the day. Nausea or vomiting did not occur. At 8 weeks of gestation, the empagliflozin dose was slightly reduced to 15 mg/day. This, however, resulted in oral mucosal lesions, and the dose was increased to 20 mg/day again. From week 6 to 12 of gestation, phenprocoumon therapy was paused due to embryotoxicity of high‐dose phenprocoumon (>3 mg/day) and replaced by low molecular weight heparin (enoxaparin, Clexane®) twice daily subcutaneously. From week 13 to 34 of gestation, the patient received phenprocoumon again and was then switched back to enoxaparin. Serial ultrasound and Doppler examinations were performed to assess fetal wellbeing. Likewise, serial transthoracic echocardiographies were performed to assess patency of the mechanical valve, and coagulation profiles to monitor adequate anticoagulation. Severe hypoglycemia with a glucose concentration of about 40 mg/dl occurred three times throughout pregnancy. Otherwise, metabolic control was excellent (transaminase activities and uric acid within normal range, triglycerides <2.2 mmol/L). Total weight gain throughout pregnancy was 11 kg.

Mode of delivery was discussed with all involved specialists, and elective CS recommended. It was performed under general anesthesia in the 37th week of gestation. Intravenous administration of glucose (12.5 g of glucose per hour) was started prior to surgery and successfully prevented hypoglycemia. Blood loss was estimated at 700 ml, and no bleeding/thrombotic complications occurred under management with unfractionized heparin starting the day before CS, and prophylactic administration of oxytocin (23 IU i.v.) and misoprostol (1 mg rectally). An apparently healthy fetus was delivered (male, 2940 g, 41st centile, length 48 cm, and head circumference 34.5 cm, Apgar score at 1, 5, and 10 min of 9, 10, 10). The newborn's blood glucose concentrations were monitored for 3 days and showed no hypoglycemia. Breastfeeding was successfully initiated, and he shows normal development at his current age of 3 months. Abdominal ultrasound of the mother performed 3 months after pregnancy demonstrated hepatomegaly with no focal lesions or adenomas. Urine analysis showed no microalbuminuria, and the kidney function remained stable.

## 3 DISCUSSION

Due to advances in treatment, patients with GSD Ib nowadays survive into adulthood, and pregnancies in this patient cohort are becoming more common.^6^ However, only seven successful pregnancies in four women with GSD Ib have been reported so far.^6^, ^7^ Pregnancy‐related complications in GSD I comprise metabolic instability due to the associated increase in energy demand and hormonal adaptations,^9^ growth of liver adenomas due to increased estrogen levels, worsening of renal function with increased albuminuria, kidney stones, renal calcifications, and infections due to neutropenia and neutrophil dysfunction.^6^, ^7^, ^9^, ^10^ Furthermore, lactic acidosis as seen in patients with recurrent hypoglycemia can be associated with premature labor.^6^, ^9^

Despite a particularly high‐risk constellation in patient 2, both pregnancies were unremarkable with no major complications. Hyperemesis did not occur, however, both patients experienced unstable glucose concentrations within the first trimester. The carbohydrate demand was highest at the end of the third trimester with a short fasting tolerance requiring frequent meals during the day and several doses of cornstarch or Glycosade® during the night (every 2.5 h). Adenoma formation, worsening of the kidney function and stone formation were not observed.

Within the last 2 years, SGLT2 inhibitors such as empagliflozin have emerged as a new treatment option for neutropenia and neutrophil dysfunction in patients with GSD Ib.^5^ Data on more than 110 patients have shown that empagliflozin has a positive impact on all neutropenia and neutrophil dysfunction related symptoms.^11^ Therefore, empagliflozin is already replacing G‐CSF as the first‐line therapy for neutropenic GSD Ib patients. It is expected that early empagliflozin treatment will at least partially prevent infectious and inflammatory long‐term complications in individuals with GSD Ib, further ameliorating the prognosis of this rare disease. The use of empagliflozin in pregnancy, however, poses the question of safety. Empagliflozin is currently not approved for use in pregnancy and during breastfeeding. The same is true for G‐CSF, which according to the pregnancy and lactation labelling rule is recommended for use in pregnancy only if the potential benefit justifies the potential risk to the fetus. However, several cases of healthy offspring from pregnancies of patients with GSD Ib on G‐CSF and patients with neutropenia from other causes on G‐CSF during pregnancy have been published.^6^, ^12^, ^13^, ^14^, ^15^, ^16^, ^17^, ^18^ In contrast, data on the use of empagliflozin during pregnancy are almost missing. This is likely due to the fact that empagliflozin is approved for the treatment of diabetes type 2, a disease that usually occurs beyond the child‐bearing age. Furthermore, there are many alternative treatment options for diabetes type 2, and attending physicians will therefore recommend to stop empagliflozin in women planning pregnancy.

Animal studies have shown that empagliflozin crosses the placenta during late gestation to a very limited extent, but no direct or indirect harmful effects with respect to early embryonic development were observed.^19^ Empagliflozin administered during the period of organogenesis was not teratogenic. However, animal studies have shown potential adverse effects, especially reduced weight gain of offsprings, on postnatal development. To our knowledge, there is only one case report of a diabetes type 2 patient who conceived while on therapy with empaglifozin plus metformin and insulin degludec.^20^ In this patient, empagliflozin treatment was stopped as soon as pregnancy was diagnosed 5 weeks after the last menstruation. This mother delivered an apparently healthy child with no congenital malformations or other pathology. Our patient 2 decided to continue empagliflozin treatment after information about the potential side effects of both empagliflozin and G‐CSF according to the available scientific data. Under this treatment, no infections or inflammatory complications occurred and the neutrophil count remained within the normal range throughout pregnancy. The patient delivered an apparently healthy baby, and wound healing of the CS scar was very good. Although our experience is in accordance with the animal data and the case reported by Formoso et al.,^20^ controlled clinical trials and data on larger patient numbers are necessary to confirm the safe use of empaglifozin during pregnancy and breastfeeding. Patient 1 who already planned to become pregnant when empagliflozin treatment became available for GSD Ib decided to continue G‐CSF throughout pregnancy. Under a dose of 0.45 μg/kg/day of G‐CSF she had persistent neutropenia. Although no major infections occurred apart from one dental infection requiring antibiotic treatment, she experienced severely impaired wound healing following the CS.

Optimal perinatal management requires good planning and interdisciplinary collaboration between metabolic physicians and obstetricians to ensure fetal and maternal wellbeing. To prevent hypoglycemia during delivery, a high glucose‐electrolyte infusion should be administered, and blood glucose as well as lactate concentrations need to be closely monitored during labour. Breast feeding is often challenging for patients with GSD I due to the high energy demand.

## 4 CONCLUSIONS

The prognosis of GSD Ib is constantly improving, and we expect that SGLT2 inhibitors will become the first‐line therapy for GSD Ib‐associated neutropenia and neutrophil dysfunction. This treatment is oral, effective, well tolerated and has proven to be beneficial in patients with GSD1b; however, the effect of the drug in pregnancy remains limited to an *n* = 1, indicating the need for close monitoring for hypoglycemia, urinary tract infections, hydration status, and renal function. The potential benefits and side effects should be discussed with the patients prior to initiation until more evidence is available. Patients with GSD1b, who were not on the drug prior to pregnancy, or who have renal disease or repeated urinary tract infections will need careful individualized consideration. Hormonal adaptations during pregnancy and the increased energy demand associated with pregnancy pose women with GSD Ib at risk of metabolic derangement and instability. Careful monitoring during pregnancy and good interdisciplinary planning of delivery is necessary to minimize the risk for both mother and child. Our experiences with the use of empagliflozin in pregnancy are encouraging, however, further studies are warranted to evaluate the safety of this drug during pregnancy and lactation.

## FUNDING INFORMATION

Not applicable.

## CONFLICT OF INTEREST

The authors declare that they have no competing interests.

## CONSENT FOR PUBLICATION

Both patients gave their informed consent for the publication of this case report.

## ETHICS STATEMENT

Not applicable.

## Data Availability

Not applicable.
